# Palliative care models for patients living with advanced cancer: a narrative review for the emergency department clinician

**DOI:** 10.1186/s44201-022-00010-9

**Published:** 2022-08-05

**Authors:** Corita R. Grudzen, Paige C. Barker, Jason J. Bischof, Allison M. Cuthel, Eric D. Isaacs, Lauren T. Southerland, Rebecca L. Yamarik

**Affiliations:** 1 Ronald O. Perelman Department of Emergency Medicine, New York University Grossman School of Medicine, New York, NY 10016 USA; 2 Department of Medicine, University of Florida Health, Gainesville, FL USA; 3 Department of Emergency Medicine, The Ohio State University Wexner Medical Center, Columbus, OH USA; 4 Department of Emergency Medicine, Zuckerberg San Francisco General Hospital, University of California San Francisco, San Francisco, CA USA; 5 Department of Medicine, Tibor Rubin Long Beach Veteran Affairs, Long Beach, CA USA

**Keywords:** Emeregency medicine, Advanced cancer, Models of care, Palliative care

## Abstract

Eighty-one percent of persons living with cancer have an emergency department (ED) visit within the last 6 months of life. Many cancer patients in the ED are at an advanced stage with high symptom burden and complex needs, and over half is admitted to an inpatient setting. Innovative models of care have been developed to provide high quality, ambulatory, and home-based care to persons living with serious, life-limiting illness, such as advanced cancer. New care models can be divided into a number of categories based on either prognosis (e.g., greater than or less than 6 months), or level of care (e.g., lower versus higher intensity needs, such as intravenous pain/nausea medication or frequent monitoring), and goals of care (e.g., cancer-directed treatment versus symptom-focused care only). We performed a narrative review to (1) compare models of care for seriously ill cancer patients in the ED and (2) examine factors that may hasten or impede wider dissemination of these models.

## Introduction

Within the last 6 months of life, 81% of persons living with cancer have an emergency department (ED) visit [1]. The majority of persons with cancer in the ED are at an advanced stage with high symptom burden and complex needs [2]. From the ED, over half is admitted to an inpatient setting, and they have high subsequent healthcare utilization and increased mortality in the month following their ED visit [3, 4]. For emergency clinicians, the path of least resistance for the care of these complicated patients is acute care admission. Hospitalization is not always the best solution however, as it comes with potential harms such as iatrogenic illness, deconditioning, and (during the pandemic) reduced visitation from loved ones and caregivers [5]. On the other hand, discharge home or to hospice requires significantly more planning and coordination of services from the ED team. Hospice coordination in particular can be difficult [6, 7].

Innovative models of care have been developed to provide high-quality, ambulatory, and home-based care to persons living with serious, life-limiting illness, such as advanced cancer. These include both face-to-face and telehealth-based visits and/or monitoring. While these programs are growing in number and reach, they are rarely known or accessible to emergency clinicians [8]. The growing integration and cross-training in emergency and hospice and palliative medicine are an opportunity to enhance collaboration between acute and supportive care services.

Multiple care models exist for seriously ill cancer patients, including inpatient and outpatient palliative care, telehealth and home-based palliative care (HBPC), home and inpatient hospice, and palliative care provided through integrated mobile technologies. These care models can be divided into categories based on level of care (e.g., lower versus higher intensity needs, such as intravenous pain/nausea medication or frequent monitoring), and goals of care (e.g., cancer-directed treatment versus symptom-focused care only). While use cases exist for all of these models, there are barriers specific to each model, therefore hindering widespread adoption. Barriers include the following: technical requirements, training required, reimbursement, evidence base, and patient or family burden (financial or otherwise) [9, 10]. For example, while discharge to home hospice can help avoid an admission in a patient with serious illness, the caregiving burden on family is substantial as compared to an inpatient stay [9]. While hospice requires patients to forgo disease-directed treatments and meet disease-specified prognostic guidelines, palliative care providers often see patients with similar prognoses that are still pursuing cancer-directed therapies [9]. Diagnostic uncertainty and difficulties with prognostic evaluation can be a barrier to hospice admission as well as patient/family reluctance to “give up” on cancer treatment [11] The median length of stay on hospice is 18 days, very late in the disease course, and 25% of patients on hospice lives <  = 5 days [12].

We performed a narrative review to understand the evidence base for each of these models of care, as well as factors that may hasten or impede wider dissemination.

## Methods

United States (US)-based physician content experts board certified in emergency medicine and/or hospice and palliative medicine each reviewed the literature on their respective care model. They all practice in academic medical centers and have between 5 and 28 years of clinical experience. The content experts convened first via videoconference to develop a consensus-based approach in defining models of care in advanced cancer patients who visit the ED. The list of models was confirmed, and then, relevant literature on each model was reviewed between March 10, 2022 and April 18, 2022. Each expert curated their literature review on each model of care based on the following themes: evidence base, benefits and barriers, care intensity, reimbursement, healthcare team composition, technical requirements, and patient/family burden. PubMed was the primary search engine tool. If no information was found in the academic literature, other reputable sources were used (e.g., governmental, organizational websites). Search terms all included emergency department and advanced cancer in addition to the model of care (e.g., inpatient palliative care, home-based palliative care). Two content experts performed each literature search to ensure all the appropriate references were captured for each care model. The Scale for the Assessment of Narrative Review Articles (SANRA) was completed [13].

### Models of Care

#### Inpatient palliative care

##### Description and team composition

Of all the varied methods of palliative care delivery, inpatient consult service is the most established with strong evidence for efficacy and cost savings [14]. In 2019, 72% of hospitals with 50 or more beds had a palliative care team, up from 67% in 2015, 53% in 2008, and 7% in 2001 [15]. The makeup of inpatient palliative care teams varies widely across hospitals in the USA but can include clinicians, nurses, social workers (SW), chaplains, and pharmacists [16].

##### Benefits

Studies of the integration of oncology and palliative care show improved survival and symptom control, less anxiety and depression, reduced use of futile chemotherapy at the end of life, better family satisfaction and quality of life, and improved use of healthcare resources [17–20]. Studies have shown reduced costs for cancer patients receiving inpatient palliative care [21]. Utilization of inpatient palliative care consults increased for patients with primary brain malignancies from 2.3% in 2007 to 11.3% in 2016, indicating a substantial increase but still far below the number of patients who could benefit from services to manage symptoms and improve quality of life [22]. Studies have shown that oncology readmission rates can be reduced by palliative care consultation, mainly due to discharge to hospice [23]. Palliative care consultation triggered by the emergency department has been shown to improve quality of life [24].

##### Barriers

Most patients are referred to inpatient palliative care very late in their illness course, often within the final weeks of life [25]. Earlier involvement of inpatient palliative care would improve care quality at the end of life [26]. One perceived barrier is that patients and families’ negatively view palliative care, equating it with death and hopelessness; this can be overcome with improved explanation by oncologists and other non-palliative care clinicians [27].

### Outpatient palliative care

#### Description and team composition

Outpatient palliative care is delivered in a variety of clinical settings including embedded within oncology clinics (in the same physical location), as well as in standalone clinics [28–30]. Currently, there are no randomized controlled trials that compare the different clinic models. However, existing studies suggested that embedded clinics had earlier referrals and greater frequency of visits [29]. Many experts feel embedded models increase collaboration with oncologists and improve convenience for the patients [31].

In addition to differences in physical location, the composition of outpatient palliative teams varies and may include a single physician or an interdisciplinary team (IDT) of advanced practice clinicians, nurses, social workers, psychologists, and/or chaplains [29]. The gold standard includes an interdisciplinary team; however, this is not feasible in some institutions. Longitudinal visits with outpatient palliative care along the course of illness allow for opportunities for rapport building, advance care planning, counseling and education, optimization of symptoms, and crisis prevention [29, 31]. Initial encounters typically last about an hour, and subsequent visits are most commonly conducted monthly [32].

#### Benefits

Outpatient palliative care has been the gold standard for early palliative care involvement in patients with advanced cancer since the landmark trial by Temel et al. in 2010. In this study, patients with advanced non-small cell lung cancer were randomized to receive early palliative care within 8 weeks of diagnosis integrated with standard oncologic care versus standard oncologic care alone. Compare to those receiving standard care alone, patients receiving early palliative care had improved quality of life, less depression, and a 3-month survival advantage [19]. Following this study in 2012, the American Society of Clinical Oncology (ASCO) recommended that palliative care be integrated into oncology services early in the disease course and concurrently with other life-prolonging therapies [33]. Subsequently, multiple studies have confirmed the benefits of outpatient palliative care showing decreased depression [34] and improved quality of life [18, 34–36], symptom burden [35–37], satisfaction with care [37–40], communication about end-of-life care preferences [34], survival [35, 36, 41], and healthcare utilization [37, 42].

#### Barriers

Despite the benefits, multiple studies indicate that palliative care access remains limited and delayed even in highly resourced institutions [43]. A survey of cancer centers published in 2020 showed that National Cancer Institute (NCI) designated cancer centers have improved access to outpatient palliative care with 95% now having outpatient palliative care; however, only 40% of non-NCI designated centers had access to outpatient palliative care [44]. Moreover, a study in California showed that outpatient palliative care capacity for oncology patients was only 24% of the estimated need [31]. Despite access being limited, the growing body of literature that demonstrates improved outcomes has led to a robust growth of outpatient palliative care over the last decade [29, 44]. Additional barriers include leaving home and travel to clinic and cost of co-payment. Reimbursement for outpatient palliative care is established and mirrors similar outpatient specialties, such as standard outpatient billing codes in a fee-for-service model of reimbursement [10].

### Telehealth-based palliative care

#### Description and team composition

Telehealth has been defined by the US Health Resources and Service Administration as “the use of electronic information and telecommunications technologies to support long-distance clinical health care, patient and professional health-related education, public health and health administration” [45]. Due to provider shortages and the challenges inherent in patients with serious illness traveling to clinic visits, traditional models of outpatient and home-based hospice and palliative care, where specially trained physicians, advanced practices providers, and other interdisciplinary team (IDT) members provide office-based care, are increasingly unable to meet the needs of the growing number of older adults with serious, life-limiting illnesses, such as cancer [46, 47]. Telehealth may augment and extend traditional clinic visits utilizing remote symptom monitoring of patients and caregivers using cellphone applications and virtual visits by clinicians [48, 49].

#### Benefits

For palliative patients with high symptom burden and poor functional status, decreasing the burden of travel to clinic has real benefit. For common cancer symptoms such as pain, anxiety, depression, and fatigue, the telehealth model is an effective way to monitor and treat them [50]. Telephonic augmentation can improve both symptom management as well as promote earlier hospice referrals [51]. Nurse-led telephonic programs and tele-palliative care models are in early stages of development but have the potential to widen the ability of palliative care to provide flexible, patient-centered care to patients with advanced cancer [52]. Two reviews of telephonic models in cancer care have shown benefit in symptom management in cancer, particularly related to anxiety, mental and emotional health, and fatigue [53–55]. The PRO-TECT randomized trial enrolled 1191 patients to either weekly internet or automated telephone surveys regarding symptoms which triggered automatic alerts to their oncology providers. Three-month follow-up surveys showed an improvement in physician function, symptom control and quality of life in the survey group [56]. 

#### Barriers

The main barrier inherent to nurse-led telephonic care is the inability of nurses to prescribe medications. This requires a telephonic, nurse-led program to actively engage with primary care clinicians and specialists which can be challenging, particularly if the nurse and provider do not work within the same health system. Programs may also struggle with coordinating care with primary providers and specialists [57, 58]. Additionally, patient identification, structure, and operational aspects of these programs have not been well described, making program replication challenging. One study of a nurse-led telephonic program for patients with lung cancer at any stage in the Veterans Affairs system did not show benefit, calling into question which patients may benefit from early access to services [59]. A large Patient-Centered Outcome Research Institute (PCORI) funded trial is currently underway to answer these questions as it compares nurse-led telephonic care to traditional clinic-based palliative care for patients with serious illness randomized after an ED visit [52]. Reimbursement is undeveloped, although some programs have been funded by insurers and/or health systems, and the Centers for Medicare & Medicaid Services (CMS) provided reimbursement for virtual physician telehealth during the COVID-19 pandemic [59].

### Home-based palliative care

#### Description and team composition

Due to increasing number of homebound adults, more attention has been paid to home-based palliative care (HBPC). In 2011, a study found that about two million US adults are homebound, and another 4.6 million are semi-homebound [60]. The 1-year mortality rate for homebound patients is 40% while 20% for semi-homebound, compared to 6% for non-homebound [61]. HBPC programs vary greatly in composition and can include nurses, nurse practitioners, social workers, physicians, chaplains, pharmacists, physical and occupational therapists, and health aides [62]. They are typically lower touch than hospice programs, offering perhaps a monthly in-person visit supplemented by telephonic or telehealth care compared to weekly visits at minimum for patients on hospice. The early adopters of this model of care have typically been large healthcare organizations with shared cost, closed health systems such as the Kaiser Permanente, insurance companies, hospice agencies, and stand-alone companies partnering with insurers [61, 63–65].

#### Benefits

As with all palliative care models, HBPC can assist patients with > 6-month prognosis who do not qualify for the Medicare hospice benefit or those who wish to continue receiving life-prolonging therapies unavailable to those on hospice. Seriously ill patients may struggle with transport to traditional palliative care clinics, and home-based programs are designed to bring comprehensive palliative care to patients in their home to improve quality of life and lower costs, mainly by avoiding hospital admissions [62]. HBPC care is still early in its development, but it has demonstrated reductions in ED visits, intensive care unit admission, hospitalization, and nursing home admissions for patients at the end of life [66, 67]. Improvement in costs is seen during the last month of life as it leads to more frequent death in the home and use of hospice [61, 68]. HBPC has been shown to improve symptoms such as pain, constipation, depression, fatigue, dyspnea, and anxiety [69]. It has also been shown to improve concordance between actual and preferred location of death [70].

#### Barriers

A national survey of HBPC organizations found a substantial lack of standardization of practice guidelines, oversight, and a lack of payment structures [71, 72]. Sustainable financing methods are lacking, and HBPC program design is not standardized to the same degree as hospice programs. These barriers contribute to a lack of patient, family, and provider understanding of HBPC, leading to challenges in referral and enrollment [71, 73]. Reimbursement is still developing for HBPC. Programs have been funded on a per-member per-month method by insurers and health systems as well as via CMS demonstration projects [74, 75].

### Community paramedicine (CP)

#### Description and team composition

Mobile integrated healthcare (MIH) is a broad term including healthcare services delivered by many types of health professionals [76]. Community paramedicine (CP), while part of MIH, involves paramedics working to support and enrich existing programs and care plans with the goal of improving care for patients while decreasing ED visits [77]. This model of CP is still evolving, with several pilot programs working towards different aims depending on geographic location and regulatory body [78–81]. Some programs require staff, such as paramedics and/or emergency medical technicians (EMTs), to receive additional training, education, and skills to care for patients at home or in alternate destinations (e.g., primary care) [76]. Select CP pilot programs are connected with hospitals or care organizations and check on patients after hospital discharge or continue a specialized care plan while respecting patient wishes to be at home and avoid the discomfort of an ED visit according to patient preferences [82, 83].

#### Benefits

CP programs offer treatment to persons living with cancer suffering from burdensome symptoms or associated treatment toxicities that may prevent an ED visit. CP can deliver time-sensitive therapies such as rehydration and nausea control and even lab services [84]. They may provide a bridge to other health professionals, via telehealth or other forms of communication [77, 85–89]. For example, patients under hospice care, for example, may receive pain medication via MIH or CP programs while waiting for the arrival of a hospice nurse. In addition, many CP programs perform wellness checks, assess and address other at-home needs, and conduct social interventions impacting the future health and wellness of vulnerable populations [81, 82].

CP leverages the strengths of the out-of-hospital care system, previously focused on patient care and transportation, while honoring patient wishes and patient-centered care. Transportation to the ED is not always the best option for advanced cancer patients who may need only rehydration and/or labs and can be spared a lengthy, often uncomfortable ED visit [90, 91]. Paramedics can efficiently and effectively provide the care patients want or need and deliver care where patients choose to receive it. The infrastructure distributing paramedics geographically to serve community settings from rural to urban during all hours of the day and night already exists and operates under medical guidance as part of an organized system of care [84]. Moreover, paramedics are already trained to perform patient assessments outside the hospital, and generally, the public already maintains a high level of trust and appreciation for this group of clinicians.

#### Barriers

Due to the fact many CP programs are in pilot phase, evaluation of the impact of these programs is still in its infancy [92]. In terms of sustainability, CMS is currently piloting a new model Emergency Triage, Treat, and Transport (ET3) [93]. This voluntary, 5-year payment model aims to provide greater flexibility and reimbursement for alternative destinations or initiation and facilitation of treatment at the scene of an emergency response or via telehealth. The goal of the ET3 model is improved quality of care with reduced system costs through decreased ED transports and preventable hospitalizations resulting from those transports [93].

### Hospice

#### Description and team composition

Hospice most commonly occurs at home with family providing the day-to-day care (called routine care), while the hospice agency provides medications, durable medical equipment, and frequent visits by a nurse-led interdisciplinary team (nurse, social worker, chaplain, home health aide, and physician) [94]. Nurse visits occur from once a week for stable patients to as often as 3 to 4 times a week for high-need patients, but a patient must have a caregiver in the home to receive home hospice. Hospice can also occur in a nursing facility if there is no home or caregiver. Patients with difficult-to-control symptoms requiring parental medications or closer monitoring can receive higher levels of hospice care with a 24-h nurse in the home (continuous care (CC)) to manage symptoms with frequent assessments/medication changes or in a skilled nursing facility (general inpatient care (GIP)) [95]. Medicare reimburses hospices with a much higher daily rate for skilled nursing facility care, but this care is scrutinized for overuse and is typically used for a few days for patients with out-of-control symptoms [96, 97]. Some hospitals have beds dedicated to hospice care that are managed by a local hospice agency, allowing the ED to admit directly to hospice within the hospital for high-need patients [98].

#### Benefits

Hospice is most beneficial if patients live at least a few weeks to get the full benefit of the service. Moving hospice enrollment upstream is important, since length of stay on hospice has dropped over the last 15 years to a median of 18 days [99, 100]. The ED is where patients present during a transition or a crisis and can be the ideal place to refer to hospice and avoid an unnecessary admission. Hospice agencies can admit patients from the ED directly to routine home care or general inpatient (GIP)/continuous care (CC) if they have high symptom burden [94]. If a hospital has a GIP unit inside their hospital, patients can be referred there if they meet criteria.

EDs and hospitals can build relationships with reliable, high-quality local hospice agencies to achieve direct referral to hospice from the ED. Some hospitals with inpatient GIP units housed within their hospital can expedite referrals of high symptom burden patients without admission to the medical ward. However, if a hospital does not have an inpatient hospice GIP unit, the hospice agency may be able to move them from the ED to a nursing facility where GIP is available [101]. This requires significant coordination and a strong relationship with local hospice agencies. An ED observation unit can be helpful for these patients to give more time to facilitate hospice placement [101]. ED or palliative care-based case managers or social workers familiar with hospice are integral to an effective transition to hospice care [102]. Standardizing ED processes and referral mechanisms may also facilitate the ED to hospice transition [103]. Reimbursement for hospice is well-established through Medicare and private insurance [104, 105].

#### Barriers

Hospice services are underutilized and frequently occur too late in the disease course to spare patients from burdensome care in the final weeks of life [106]. Patients are required to forgo life-sustaining therapies such as chemotherapy, radiation, and future hospitalization when they enroll in hospice which may delay hospice admission [107]. Patients and their clinicians often have a discordant understanding of a patient’s end-of-life goals. If a patient is on routine care on hospice and is unable to live at home due to homelessness or lack of caregiver in the home, they will need nursing home care while on hospice. The nursing home is not paid for by hospice which can be a financial barrier, if the patient is unable to pay out of pocket. Medicaid will pay for the nursing home stay if a patient is on routine hospice care, but many patients are not enrolled even though they may qualify, and the enrollment process can be lengthy, preventing enrollment on hospice from the ED [9, 108, 109].

## Implications for emergency providers

**Fig. 1:**
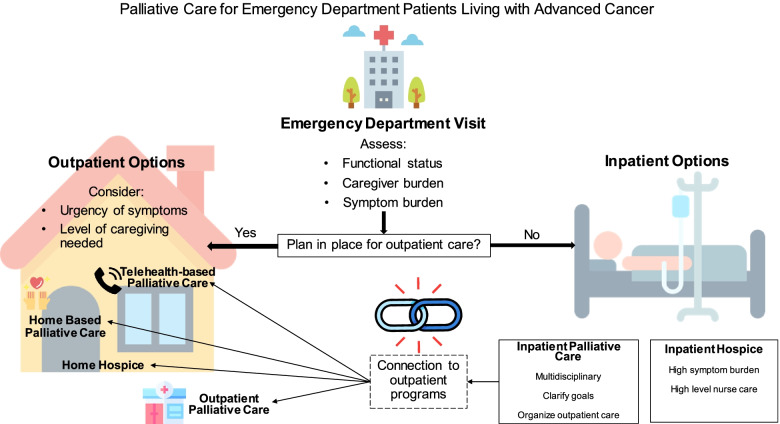
Palliative care for emergency department patients living with advanced cancer

Patients with advanced cancer face myriad challenges in the final years of life. These challenges frequently result in ED visit and subsequent inpatient admission. Many new models of care exist and are being developed to attempt to address these challenges as a way to prevent some ED visits and avoid hospital admission altogether. The forms of palliative and hospice care discussed in this paper have variable penetration in different areas of the country as well as differences between health systems and insurers. Table 1 provides a snapshot summary of the characteristics of each model of care, while Fig. 1 depicts how they intersect. EM providers can educate themselves as to what palliative care and hospice programs are active in their area, recognizing not all services are available within all healthcare systems. Understanding what programs cancer patients in the ED already have at home, in addition to what they might be eligible, may help prevent future ED visits.

**Table 1:** Characteristics of models of care in advanced cancer, regardless of prognosis

Service	Intensity	Reimbursement	Evidence base	Technical requirements	Patient/family burden
Inpatient palliative care	High	Established	Strong	Low	Low
Outpatient palliative care	Low	Established	Strong	Low	Moderate
Telehealth-based palliative care	Variable	Developing	Weak	Moderate	Variable
Home-based palliative care (HBPC)	Variable	Developing	Weak	Variable	Variable
Community paramedicine (CP)	Variable	Undeveloped	Weak	Low	Variable
General inpatient (GIP) or continuous care (CC) hospice	High	Established	Strong	Low	Low
Home hospice	Low	Established	Strong	Low	High

Intensity: level of intensity refers to level of monitoring and support provided to patient by paid healthcare team (e.g. high intensity is comparable to inpatient care) [110]. Reimbursement: reimbursement evaluated based on whether Medicare beneficiaries have complete coverage (developed), incomplete coverage (developing) or not covered (undeveloped) [104]. Evidence base: model of care has been tested in randomized controlled trials and shown benefits to patient-centered outcomes [111]. Technical requirements: defined as whether care requires a telehealth device, software and/or Internet connection [112]. Patient/family burden: the level of family burden was defined as models in which a caregiver must provide the majority (high burden) or the minority (low burden) or care to the patient under the supervision of a healthcare team [111, 113]

## Conclusion

The breadth of acute and supportive care models to support persons living with advanced cancer has grown substantially. Most hospitals have inpatient palliative care consult services to assist patients who are admitted. Hospital admissions can be avoided by hospice admission occurring directly from the ED for patients with hospice goals. HBPC, outpatient palliative care, CP, and telehealth support patients who still seek disease-modifying therapies but need additional symptom management and psychosocial supports to prevent ED admissions. ED providers now have multiple tools they can leverage to improve care for cancer patients and potentially avoid ED transport and hospital admission. Advances in technology and retraining clinicians to work in new ways and in new settings have spurred innovation and the ways in which clinicians can care for patients in settings most aligned with their values and preferences. Meanwhile, reimbursement mechanisms have not always kept pace with the proliferation of care models, presenting a barrier to more widespread adoption of these practices.
